# Full-term low birth weight infants have differentially hypermethylated DNA related to immune system and organ growth: a comparison with full-term normal birth weight infants

**DOI:** 10.1186/s13104-020-04961-2

**Published:** 2020-04-03

**Authors:** Ikuyo Hayashi, Ken Yamaguchi, Masahiro Sumitomo, Kenji Takakura, Narumi Nagai, Naoki Sakane

**Affiliations:** 1 Clinical Research Institute, National Hospital Organization Kyoto Medical Center, 1-1, Mukaihata-cho, Fukakusa, Fushimi-ku, Kyoto, 612-8551 Japan; 2 Department of Obstetrics and Gynecology, National Hospital Organization Kyoto Medical Center, Kyoto, Japan; 3 Department of Gynecology and Obstetrics, Kyoto University Graduate School of Medicine, Kyoto, Japan; 4 Tajima KOUNOTORI Perinatal Medical Center, Toyooka Hospital, Toyooka, Japan; 5 Laboratory of Nutrition Education and Nutritional Physiology, Graduate School of Human Science and Environment, University of Hyogo, Himeji, Japan

**Keywords:** Low birth weight, Term infant, Gene, DNA methylation, Gene ontology (GO), Immune system

## Abstract

**Objective:**

Low birth weight (LBW) is a major public health issue as it increases the risk of noncommunicable diseases throughout life. However, the genome-wide DNA methylation patterns of full-term LBW infants (FT-LBWs) are still unclear. This exploratory study aimed to analyze the DNA methylation differences in FT-LBWs compared with those in full-term normal birth weight infants (FT-NBWs) whose mothers were nonsmokers and had no complications. Initially, 702 Japanese women with singleton pregnancies were recruited. Of these, four FT-LBWs and five FT-NBWs were selected as references for DNA methylation analysis, and 862,260 CpGs were assessed using Illumina Infinium MethylationEPIC BeadChip. Gene ontology enrichment analysis was performed using DAVID v6.8 software to identify the biological functions of hyper- and hypomethylated DNA in FT-LBWs.

**Results:**

483 hyper-differentially methylated genes (DMGs) and 35 hypo-DMGs were identified in FT-LBW promoter regions. Hyper-DMGs were annotated to 11 biological processes; “macrophage differentiation” (e.g., *CASP8*), “apoptotic mitochondrial changes” (e.g., *BH3*), “nucleotide-excision repair” (e.g., *HUS1*), and “negative regulation of inflammatory response” (e.g., *NLRP12* and *SHARPIN*). *EREG* was classified into “ovarian cumulus expansion” within the “organism growth and organization” category. Our data imply that LBW might be associated with epigenetic modifications, which regulate the immune system and cell maturation.

## Introduction

Low birth weight (LBW) is widely known to be associated with increased rates of hypertension, diabetes, obesity, stroke, and coronary heart disease in later life [1, 2]. The Developmental Origins Health and Disease (DOHaD) hypothesis suggests that chronic conditions and diseases in later life result from “prenatal programming.” In Japan, the prevalence of LBW has reached to approximately 10% in 2017, which is the highest level in the developing countries [3]. Therefore, this hypothesis, which explains the effects of in utero environment in early life, can help us to understand the likely health of the LBW later life. However, to the best of our knowledge, there are no reports of genome-wide methylation studies in LBW full-term infants (FT-LBWs).

Several fetal genome-wide DNA methylation studies have analyzed preterm LBW infants [4–6]. One such study confirmed by gene enrichment analysis that some biological processes, including neuron differentiation and nervous system development, are associated with gestational age, and reported that gestational length was more highly correlated with DNA methylation than birth weight [5]. Another study of preterm infants attempted to clarify the methylation characteristics of a large cohort [6]. Therefore, previous DNA methylation studies may have captured DNA methylation differences associated with shorter gestation term. Additionally, methylation studies in developed countries have increasingly focused on the effects of higher maternal nutrient intake and greater maternal body mass. There are many reports of specific gene methylation and expression; for example, the expression of *IGF2* (insulin-like growth factor 2) is regulated by maternal nutrient intake during fetal development [7–9].

The aim of the present exploratory study was to compare genome-wide DNA methylation between FT-LBWs and normal birth weight full-term infants (FT-NBWs) born from non-smoking mothers with no complications during pregnancy. These mother-infant pairs were strictly excluded to control the following conditions reported to affect birth weight and DNA methylation: hypertensive disorder of pregnancy (HDP) [10, 11]; gestational diabetes mellitus (GDM) [12–14]; maternal infections [15, 16]; tumors [17, 18]; mental disorders [19, 20] and smoking [21, 22]. Ultimately, the maternal backgrounds of FT-LBWs were characterized by lower pre-pregnancy body mass index (BMI). This is the first genome-wide study to investigate DNA methylation patterns in FT-LBWs without the effects of maternal complications and smoking.

## Main text

### Methods

#### Study participants

Initial participants in the present study consisted of 702 Japanese women aged ≥ 20 years who gave birth to a single child between November 2015 and April 2018. Maternal and fetal information was ascertained from medical and prenatal records. The following data was collected: maternal age, previous pregnancies (i.e., primipara or multipara), medical history (e.g., infections, tumors, hypertension, diabetes, and mental disorders), HDP, GDM, smoking, maternal height, pre-pregnancy body weight, gestational weight gain (GWG), fetal gender, gestational age, and birth weight.

The flow chart of this cohort is shown in Additional file 1: Figure S1. Of the 702 subjects, considering the effect on fetal DNA methylation, mothers who delivered at preterm, with complications, and with smoking habit in the preconception period and/or during pregnancy were excluded.

After excluding these mothers, the data of 386 mother-fetal pairs were available for DNA methylation analysis. Overall, FT-LBW (defined as a birth weight < 2500 g, born at 37–41 weeks of gestation) was 25 subjects. Among these subjects, propensity score was matched for infant gender, maternal age, and primipara/multipara status. Subsequently, by randomized selection according to the median birth weight (3019 g), four FT-LBWs and five FT-NBWs were used as references for analysis.

#### Genome-wide DNA methylation analysis

Umbilical cord blood samples were collected at birth and frozen at − 80 °C until DNA extraction. DNeasy Blood and Tissue Kits (Qiagen) were used to extract DNA from the white cell fraction of cord blood and purified.

For each subject, 865,918 CpG sites across the genome were interrogated using the Illumina Infinium Human Methylation BeadChip (Illumina, USA) [23]. Briefly, 1 µg DNA was converted with sodium bisulfite, amplified, fragmented, and hybridized according to the manufacturer’s instructions. After implementing bias adjustment by the beta-mixture quantile normalization method, 862,260 CpGs were used for analysis. *β*-values were converted into *M*-values (log_2_ ratio of *β*-value) to account for heteroscedasticity. Differences in methylation in promoter regions relative to UCSC reference sequences were identified between FT-LBW and FT-NBW. A Benjamini and Hochberg false discovery rate control was applied. DNA methylation differences were selected based on log_2_ ratio of *β*-value differences ≥ 0.6.

#### Gene ontology functional enrichment analysis

We performed functional enrichment analysis using the Database for Annotation, Visualization and Integrated Discovery v6.8 (https://david.ncifcrf.gov/) to identify the biological functions of hyper- and hypomethylated DNA [24]. This approach evaluates DNA methylation data in terms of categories of gene function rather than individual genes. Genes were ranked by magnitude of correlation with a class distinction in the GOTERM_BP algorithm, and an enrichment score was calculated. Significance was defined as *P* < 0.05.

#### Statistical analysis

Participants’ characteristics and baseline data are presented as *n* (%) for categorical variables or mean ± standard deviation for continuous variables. The Chi square test (categorical data) and the student’s *t* test (continuous data) in SPSS (Institute, version 23, IBM) was performed to compare characteristics between FT-LBW and FT-NBW groups. Significance was defined as *P* < 0.05.

### Results

#### The characteristics of participants

The number of patients excluded and the characteristics of participants included for DNA methylation analysis are presented in Additional files 1 and 2, respectively. After the excluding the participants who had delivered preterm, those with complications, and/or those with a habit of smoking, 25 FT-LBW and 361 FT-NBW were finally enrolled in the study (Table 1). Birth weight (*P *< 0.001), gestational age (*P *< 0.001) and infant gender (*P* = 0.023) were significantly different between FT-LBWs and FT-NBWs. The pre-pregnancy BMI of FT-LBW mothers was also significantly lower than that of FT-NBW mothers (*P *= 0.044). There were no statistically significant differences in maternal age, GWG, socioeconomic status, household income, maternal educational level, or alcohol consumption between groups. None of the participants had used any drugs in the conception period and during pregnancy. The adjusted odds ratio (aOR) of pre-pregnancy BMI was significantly associated with FT-LBW after adjustment for maternal age and GWG (aOR: 0.77; 95% confidence interval: 0.63–0.93; *P* = 0.007).

**Table 1:** Characteristics of participants

	FT-NBW infants	FT-LBW infants	*P* value
*N*	361	25	
Age, years	31.8 ± 5.1 (20–49)	33.8 ± 5.7 (20–44)	0.06
Primipara	180 (49.9)	10 (40.0)	0.34
Height, cm	158.6 ± 5.8 (145.0–175.0)	156.4 ± 4.3 (148.0–165.0)	0.07
Pre-pregnancy body mass index, kg/m^2^	21.0 ± 2.9 (14.7–37.7)	19.7 ± 2.1 (15.2–24.0)	0.044
Gestational weight gain, kg	10.0 ± 4.0 (− 3.7–21.0)	8.6 ± 3.7 (− 1.4–15.0)	0.08
Household income, yen			
< 2 million	11 (3.0)	1 (4.0)	0.98
2–6 million	211 (58.4)	15 (60.0)	
≥ 6 million	114 (31.6)	7 (28.0)	
Unknown	25 (6.9)	2 (8.0)	
Educational level			
Junior high school	6 (1.7)	1 (4.0)	0.24
High school	65 (18.0)	7 (28.0)	
Some college	135 (37.4)	11 (44.0)	
University	155 (42.9)	6 (24.0)	
Alcohol drinking			
In conception, yes	100 (27.7)	4 (16.0)	0.46
During pregnancy, yes	0 (0)	0 (0)	–
Drug			
In conception, yes	0 (0)	0 (0)	–
During pregnancy, yes	0 (0)	0 (0)	–
Infants			
Gender, female	161 (44.6)	17 (68.0)	0.023
Gestational day, week	39.0 ± 1.1 (37–41)	37.8 ± 0.7 (37–39)	< 0.001
Birth weight, g	3056 ± 316 (2506–3994)	2362 ± 136 (1982–2606)	< 0.001

Data is represented as n (%) or mean ± standard deviation (max.-min.)

FT-NBW: full-term normal birth weight; FT-LBW: full-term low birth weight

*P* values were determined using Chi square test for categorical variables and independent student’s *t*-test for continuous variables. *P* < .05 was considered significant

Among participants who were included for DNA methylation analysis, the pre-pregnancy BMI of mothers and birth weight were significantly lower in FT-LBW than in FT-NBW. Less significant differences were found in maternal age, GWG, or gestational age with respect to birth weight.

#### Identification of DMGs

**Fig. 1:**
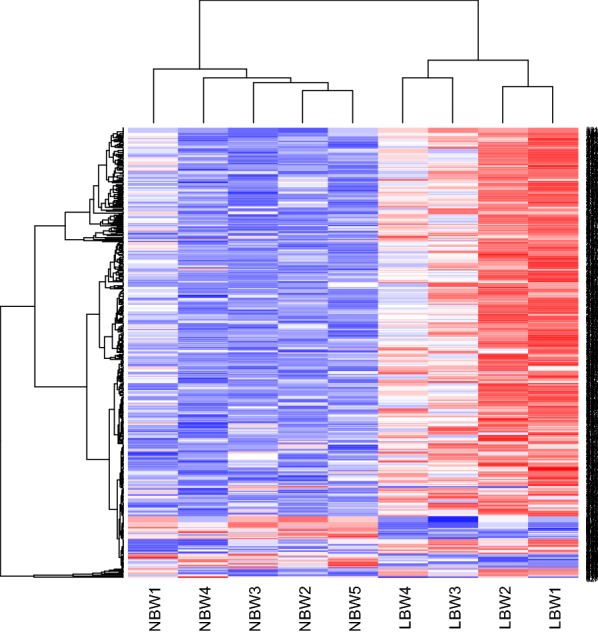
Correlation between DMGs and birth weight. Heat map illustration of the clustering of DMGs with birth weight. Red and blue colors indicate hyper- and hypomethylation, respectively. Average linkage clustering was performed on beta values based on the correlation distance between FT-LBW infants and FT-NBW infants. LBW1-LBW4: full-term low birth weight infants and NBW1-NBW5: full-term normal birth weight infants

A total of 583 hypermethylated reference sequences were determined to correspond to 483 hyper-differentially methylated genes (DMGs) and 58 hypomethylated reference sequences were determined to correspond to 35 hypo-DMGs in FT-LBW infants, compared to the FT-NBW infants (Fig. 1).

#### Enrichment analysis and functional annotation

To investigate the effect of birth weight on fetal biological processes, we performed biological enrichment analysis of hyper-DMGs and hypo-DMGs in FT-LBW using the GOTERM_BP algorithm. The 483 hyper-DMGs identified were annotated to 11 biological processes in FT-LBW infants. The functions of hypomethylated genes could not be verified. Table 2 shows GO term and genes of each enrichment biological process. Furthermore, these 11 biological processes could be further categorized as “immune system,” “DNA metabolism and repair,” and “organism growth and organization”.

**Table 2:** Biological processes and genes of differentially hypermethylation in FT-LBWs

GO term	Biological process^a^	Genes^b^	Fold enrichment	*P*
GO:0001550^e^	Ovarian cumulus expansion	*EREG*, *BMPR1B*	54.343	0.036
GO:0044356^c^	Clearance of foreign intracellular DNA by conversion of DNA cytidine to uridine	*APOBEC3A_B, APOBEC3A*	54.343	0.036
GO:0006163^d^	Purine nucleotide metabolic process	*GMPR2*, *NME/NM23*, *GUK1*	16.303	0.014
GO:0030225^c^	Macrophage differentiation	*CASP8*, *CASP10*, *BMP4*	9.057	0.042
GO:0008637^c^	Apoptotic mitochondrial changes	*BH3, IFIT2*, *AIFM2*	8.580	0.047
GO:0040018^e^	Positive regulation of multicellular organism growth	*GHR, GHRL, GPR21, GPAM*	6.211	0.026
GO:0045071^c^	Negative regulation of viral genome replication	*IFI16, ADAR, APOBEC3A_B, APOBEC3A*	5.434	0.037
GO:0006289^d^	Nucleotide-excision repair	*HUS1*, *ERCC1*, *RPA2*, *NEIL1*	5.302	0.039
GO:0050728^c^	Negative regulation of inflammatory response	*NLRP12, SHARPIN, GHRL, PBK*, *AOAH*, *SMPDL3B*	4.127	0.015
GO:0001649^e^	Osteoblast differentiation	*CREB3L1*, *FBL*, *SPP1*, *BMP4*, *ITGA11*, *ADAR*	3.135	0.043
GO:0006468^e^	Protein phosphorylation	*STRADB, BMPR1B, STRADA, PBK, BRSK1, FER, CDK8, HUS1, LATS1, COQ8B, CCNT1, CAMKK1, MATK, TGFBR1, LTK*	1.788	0.042

FT-LBW: full-term low birth weight; GO: gene ontology

^a^Biological process that were found to correspond to hypermethylated DNA in FT-LBW infants. *P* < 0.05 was considered significant

^b^Genes found to be differentially methylated in the promoter region

^c^“Immune system” category

^d^“DNA metabolism and repair” category

^e^“Organism growth and organization” category

Of these, five enrichment biological processes were classified into the “immune system” category: clearance of foreign intracellular DNA by conversion of DNA cytidine to uridine (*APOBEC3A_B* and *APOBEC3A*), macrophage differentiation (*CASP8*, *CASP10*, and *BMP4*), apoptotic mitochondrial changes (*BH3*, *IFIT2*, and *AIFM2*), negative regulation of viral genome replication (*IFI16*, *ADAR*, et al.), and negative regulation of inflammatory response (*NLRP12*, *SHARPIN*, et al.).

Two enrichment biological processes were identified into the “DNA metabolism and repair” category: purine nucleotide metabolic process (*GMPR2*, *NME/NM23*, and *GUK1*) and nucleotide-excision repair (*HUS1*, *ERCC1*, et al.).

Another four enrichment biological processes were classified into the “organism growth and organization” category: ovarian cumulus expansion (*EREG* and *BMPR1B*), positive regulation of multicellular organism growth (*GHR*, *GHRL*, et al.), osteoblast differentiation (*CREB3L1*, *FBL*, et al.), and protein phosphorylation (*STRADB*, *BMPR1B*, et al.).

### Discussion

This is the first study to compare DNA methylation between FT-LBW and -NBW infants who were born from the mothers without complications and smoking habit. Ultimately, maternal background of FT-LBWs was characterized by lower pre-pregnancy BMIs. It is important to note that participants in this study were select based on strict criteria to exclude maternal factors that may have affected DNA methylation. Our results suggest that LBW itself may be associated with epigenetic modulation of the immune system and cell maturation.

We identified the functional categories of hyper-DMGs in FT-LBWs such as macrophage differentiation, apoptotic mitochondrial changes, nucleotide-excision repair, negative regulation of inflammatory response and ovarian cumulus expansion. Interestingly, several genes annotating cell apoptosis; *CASP8 and BH3,* cell cycle progression; *HUS1* and inflammatory response; *NLRP12* and *SHARPIN* were hypermethylated in FT-LBW infants.

It is well established that *CASP8* is an important contributor in the apoptotic pathway. Sequential activation of caspases plays a central role in the execution of cell apoptosis [25]. A previous study was reported that in mice with a deletion in *CASP8* in the intestinal epithelium, inflammatory lesions spontaneously developed in the terminal ileum and mice were highly susceptible to colitis [26]. We next observed *BH3* hypermethylation in FT-LBWs. BH3-only proteins have been recognized as essential initiators of apoptosis [27]. Some studies have reported that BH3-only proteins are linked with the promotion of apoptosis in embryonic germ cells, oocytes, follicular granulosa cells, and luteal cells, as well as the regulation of oocyte number and quality in the ovary [28, 29].

We also observed increased methylation status of *HUS1* in FT-LBW infants. The protein product of this gene forms a complex with RAD9 and RAD1 and functions as a cell cycle checkpoint during replication stress, involved in response to DNA damage [30–32]. *HUS1* DNA hypermethylation could mean that error cells may perpetuate during DNA replication in FT-LBWs. Therefore, LBW could be potentially linked with impaired cell metabolism in their life.

For “negative regulation of inflammatory response” genes, the methylation status of *NLRP12* and *SHARPIN* were increased. *NLRP12* downregulates the production of inflammatory cytokines [33]. Some animal study showed that Nlrp12−/− mice were highly susceptible to colitis and colitis-associated colon cancer [34, 35]. *SHARPIN* plays a role in preventing skin inflammation by inhibiting TNFR1-induced keratinocyte apoptosis [36]. Other study reported that cutaneous inflammation in *SHARPIN*-deficient mice is autoinflammatory in nature [37]. In data from intrauterine growth restriction (IUGR) animal models which is caused due to maternal malnutrition and/or poor GWG; lower absolute immune organ weights, damaged and jagged villi, decreased villus surface areas, and a smaller number of epithelial goblet cells and lymphocytes were leaded [38]. Considering together these reports and our results, human LBWs may also have thin and weak intestinal mucosa and high sensitivity that to evoke autoinflammatory.

For the “organism growth and organization” category, the methylation status of *EREG*, which was annotated to “ovarian cumulus expansion,” was increased. The encoded protein may be involved in a wide range of biological processes including inflammation, wound healing, oocyte maturation, and cell proliferation. A previous animal study demonstrated that undernutrition during pregnancy can delay fetal follicular development in sheep [39].

In summary, some alterations in genes annotated to biological processes were identified which could be related to health problems in maternal undernutrition induced LBW infants. We would like to emphasize that it is important to select individual target attributes for preventive intervention even in a small study group and to clarify methylation patterns.

## Limitations

First, the study cohort was small since the criteria for subject selection were specific and stringent. Additional studies in other FT-LBWs cohorts are needed to verify that similar results are observed. Another limitation is that since this study was a cross-section analysis at birth, it has not been verified whether these methylation differences are actually linked to future health problems.

## Supplementary information

**Additional file 1: Figure S1.** Flow chart illustrating the data of study participants.

**Additional file 2: Table S1.** Maternal and infant characteristics in DNA methylation analysis.

## Data Availability

All data generated or analysed during this study are included in this published article and its supplementary information files.

## Abbreviations

BMI: Body mass index
CpG: Cytosine-phosphate-guanine
DMGs: Differentially methylated genes
DNA: Deoxyribose nucleic acid
DOHaD: The Developmental Origins Health and Disease
FT-: Full-term infants
GDM: Gestational diabetes mellitus
GO: Gene ontology
GWG: Gestational weight gain
HDP: Hypertensive disorder of pregnancy
LBW: Low birth weight
NBW: Normal birth weight
