# Supplementary figures and images for: Full-term low birth weight infants have differentially hypermethylated DNA related to immune system and organ growth: a comparison with full-term normal birth weight infants
Source: BMC Res Notes. 2020 Apr 3;13:199. doi: 10.1186/s13104-020-04961-2 (PMC7126402; doi:10.1186/s13104-020-04961-2)

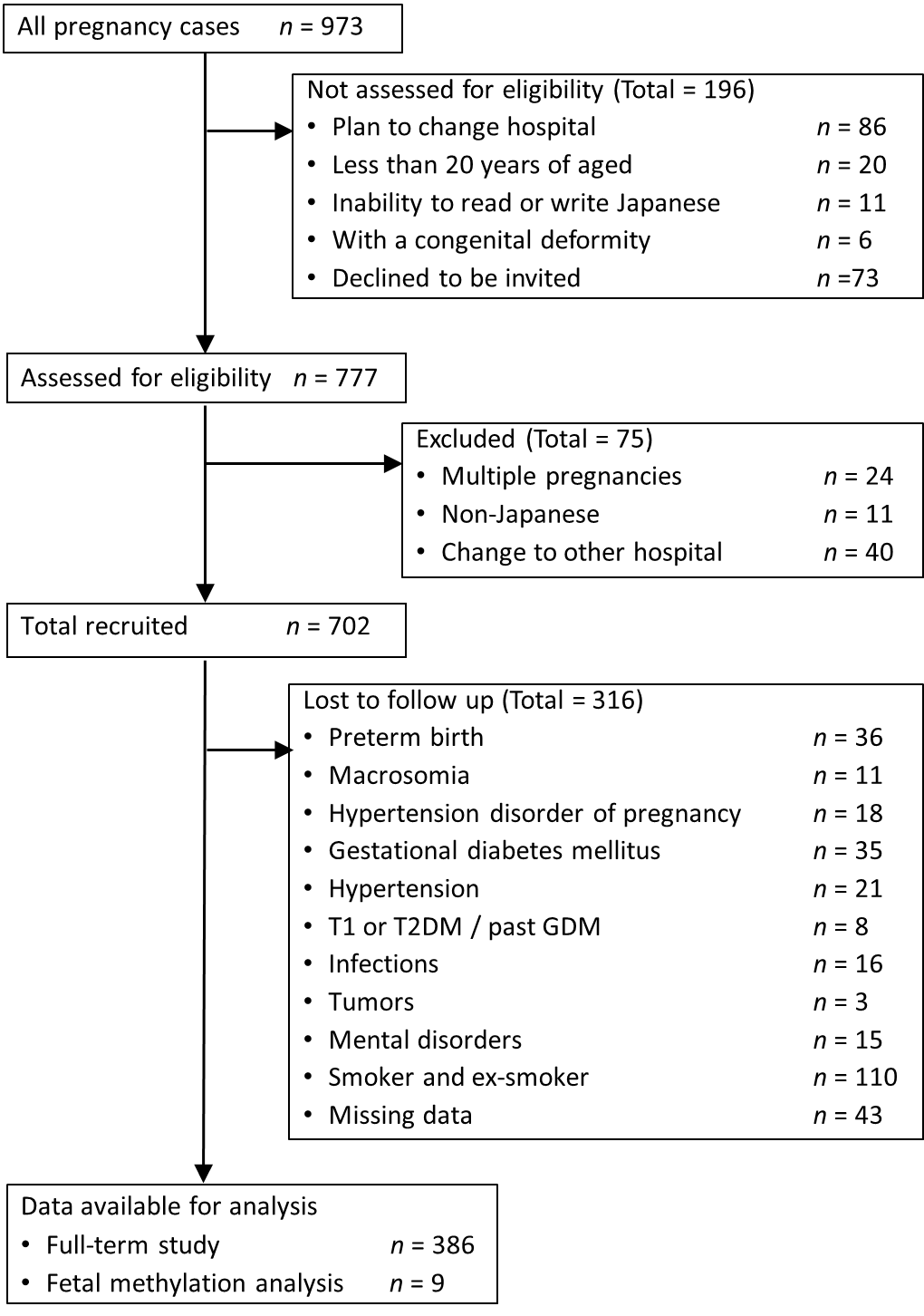

Supplement: Supplementary file 1 — Additional file 1: Figure S1. Flow chart illustrating the data of study participants. [file 13104_2020_4961_MOESM1_ESM.docx]
